# Cyanidin attenuates the apoptosis of rat nucleus pulposus cells and the degeneration of intervertebral disc via the JAK2/STAT3 signal pathway *in vitro* and *in vivo*

**DOI:** 10.1080/13880209.2022.2035773

**Published:** 2022-02-17

**Authors:** Xiaoliang Bai, Meichao Jiang, Jie Wang, Shuai Yang, Zhiwei Liu, Hongxin Zhang, Xiaojuan Zhu

**Affiliations:** a Department of Orthopaedics, Baoding NO.1 Central Hospital, Baoding, China; b Department of Spine, The Third Hospital of Hebei Medical University, Shijiazhuang, China; c Department of Geratology, Baoding NO.1 Central Hospital, Baoding, China

**Keywords:** Extracellular matrix degeneration, chloroquine, autophagy, natural polyphenolic compounds

## Abstract

**Context:**

Cyanidin has been shown to have therapeutic potential in osteoarthritis. However, it is unclear whether cyanidin prevents the progression of intervertebral disc degeneration (IVDD).

**Objective:**

This study evaluates the effects of cyanidin on IVDD *in vitro* and *in vivo*.

**Materials and methods:**

Nucleus pulposus cells (NPCs) isolated from lumbar IVD of 4-week-old male Sprague-Dawley (SD) rats were exposed to 20 ng/mL IL-1β, and then treated with different doses (0-120 µM) of cyanidin for 24 h. SD rats were classified into three groups (*n* = 8) and treated as follows: control (normal saline), IVDD (vehicle), IVDD + cyanidin (50 mg/kg). Cyanidin was administered intraperitoneally for 8 weeks.

**Results:**

The IC_50_ of cyanidin for NPCs was 94.78 µM, and cyanidin had no toxicity at concentrations up to 500 mg/kg in SD rats. Cyanidin inhibited the apoptosis of NPCs induced by IL-1β (12.73 ± 0.61% vs. 18.54 ± 0.60%), promoted collagen II (0.82-fold) and aggrecan (0.81-fold) expression, while reducing MMP-13 (1.02-fold) and ADAMTS-5 (1.40-fold) expression. Cyanidin increased the formation of autophagosomes in IL-1β-induced NPCs, and promoted LC3II/LC3I (0.83-fold) and beclin-1 (0.85-fold) expression, which could be reversed by chloroquine. Cyanidin inhibited the phosphorylation of JAK2 (0.47-fold) and STAT3 (0.53-fold) in IL-1β-induced NPCs. The effects of cyanidin could be enhanced by AG490. Furthermore, cyanidin mitigated disc degeneration in IVDD rats *in vivo*.

**Discussion and conclusions:**

Cyanidin improved the function of NPCs in IVDD by regulating the JAK2/STAT3 pathway, which may provide a novel alternative strategy for IVDD. The mechanism of cyanidin improving IVDD still needs further work for in-depth investigation.

## Introduction

Intervertebral disc degeneration (IVDD) is the most common cause of lower back pain and the basis of spinal degenerative diseases (Gao et al. 2018). As a complex pathophysiological process, IVDD is thought to be related to the imbalance of extracellular matrix (ECM) synthesis and catabolism, apoptosis, inflammation, and angiogenesis (Alpantaki et al. 2019). Due to the limited regeneration capacity, the degeneration of the lumbar intervertebral disc is difficult to be reversed. Currently, there is no effective intervention to prevent IVDD.

As the only constituent cell of the intervertebral disc nucleus pulposus, the abnormal function of nucleus pulposus cells (NPCs) seriously affects the occurrence and development of lumbar disc degeneration (Rosenzweig et al. 2017). In the nucleus pulposus, cartilage-like NPCs are coated in the extracellular matrix (ECM), which is mainly composed of collagen II and aggrecan. Excessive apoptosis of NPCs can disrupt the metabolic balance in ECM, leading to a decrease in the synthesis of matrix components, ultimately leading to IVDD (Gao et al. 2018). Inhibition of excessive apoptosis of NPCs has been considered as a potential therapeutic target for IVDD.

Autophagy is widely present in eukaryotic cells and is an evolutionarily relatively conserved lysosomal-dependent degradation pathway, which is specifically regulated (Kang et al. 2019). As a defensive measure, autophagy can respond to metabolic stress such as nutritional deprivation, oxidative stress, and hypoxia. Current research suggests that a certain degree of autophagy plays a protective role in degenerative diseases including IVDD and osteoarthritis (Li et al. 2020). As another type of cell death, the inhibitory effect of autophagy on apoptosis under certain conditions helps protect intervertebral disc cells (Chen et al. 2018), which is of significance for delaying the process of IVDD. Research on autophagy will help develop new treatments for disc degenerative diseases.

Cyanidin (cyanidin-3-glucoside chloride) is a natural polyphenolic compound extracted from grape seeds and blueberries. Cyanidin has a wide range of pharmacological activities, including anti-inflammatory, antioxidative stress, and anti-apoptotic (Acquaviva et al. 2003; Gao et al. 2013; Raj et al. 2017). In a recent study, cyanidin was found to have beneficial effects on osteoarthritis by inhibiting inflammatory responses and ECM degradation in chondrocytes (Jiang et al. 2019). Moreover, cyanidin has been reported to regulate p62 and ATG4 in renal cell carcinoma, which are related to autophagy (Liu et al. 2018). However, it is unclear whether cyanidin can prevent the progression of IVDD.

In the present study, the effect of cyanidin on the apoptosis, autophagy, and ECM degradation of degenerative NPCs was initially explored *in vitro*, and its potential mechanism of action was also investigated. We also investigated the effect of cyanidin on IVDD *in vivo* by preparing a rat model of disc degeneration. This study may provide a new direction for the treatment of IVDD.

## Materials and methods

### Animals

Male Sprague-Dawley rats (280-300 g, 4 weeks and 8 weeks) were provided by the Experimental Animal Centre of Hebei Province. Rats were housed in standard cages with an ambient temperature of 23 ± 2 °C, a humidity of 55 ± 10%, and a 12 h light/dark period. The experiment was carried out after 1 week of adaptive feeding in rats. All surgical interventions, treatments and postoperative animal care procedures were performed in strict accordance with the Animal Care and Use Committee of Baoding NO.1 Central Hospital [SYXK (Hebei Province) 2019-016].

### Isolation and culture of NPCs

Nucleus pulposus tissues were isolated from lumbar IVD of 4-week-old male SD rats. The nucleus pulposus tissues were cut into pieces of 1 mm^3^ size with ophthalmic scissors, and treated with 0.25% trypsin and type II collagenase at 37 °C for 4 h. This material was filtered through a 200 mesh strainer and centrifuged to collect NPCs. NPCs were then placed in DMEM/F12 medium (Gibco, Thermo Fisher Scientific, Inc., USA) containing 10% foetal bovine serum (FBS; Gibco) and cultured in an incubator at 37 °C and 5% CO_2_. Second-generation cells were used in the following experiments.

### Grouping of IL-1β-induced NPCs

IL-1β (20 ng/mL) was selected to treat NPCs for 24 h to induce degeneration of nucleus pulposus cell model (Deng et al. 2017). The dose of cyanidin was determined by treating IL-1β-induced NPCs with 0-120 μM cyanidin (C_21_H_21_O_11_Cl; ≥98%; A0428; Chengdu Must Bio-Technology, Chengdu, China) (Jiang et al. 2019). Cells were then divided into four groups to analyse the effects of cyanidin on ECM degradation, apoptosis, and autophagy in NPCs induced by IL-1β: control, IL-1β and IL-1β + cyanidin (25 or 50 µM) groups. To determine the role of autophagy in degenerating NPCs, IL-1β-induced NPCs were pre-treated with the autophagy inhibitor chloroquine (CQ) before cyanidin administration to prevent later autophagy. Additionally, IL-1β-induced NPCs were pre-treated with AG490 (JAK2 antagonist) before cyanidin administration to study the effects of cyanidin on JAK2/STAT3 pathway.

### Cell viability assay

The 3-(4,5-dimethylthiazol-2-yl)-2,5-diphenyltetrazolium bromide (MTT) assay was used to evaluate the activity of NPCs. NPCs cells were seeded into 96-well plates (1 × 10^4^ cells/well), incubated overnight at 37 °C, 5% CO_2_ incubator for 24 h. Then, NPCs were exposed to IL-1β, cyanidin and CQ, as described above. After indicated treatments, cells were washed with fresh medium and added to MTT solution (Sigma-Aldrich, St Louis, MO, USA) and incubated for 4 h at room temperature. After aspirating the MTT solution, the cells were incubated with 100 μL DMSO for 5 min at room temperature. The optical density values of the plate were measured at 490 nm on a Tecan Sunrise Absorbance Microplate Reader (Tecan Group, Switzerland).

### Western blot assay

Cells were lysed with protein lysis buffer (RIPA; Solarbio, Beijing, China) to extract total proteins from NPCs. Protein concentration was measured using the Bicinchoninic acid (BCA) protein concentration kit (Beyotime, Shanghai, China). Proteins were separated by sodium dodecyl sulphate polyacrylamide gel electrophoresis (SDS-PAGE) and transferred to a polyvinylidene fluoride (PVDF; Millipore, Billerica, MA, USA) membrane. The membrane was blocked for 2 h in Tris buffer containing 5% skim milk. After washing with PBS, the membrane was incubated with the corresponding primary antibody against aggrecan (1:1000; ab3778; Abcam, Cambridge, MA), collagen II (1:1000; ab188570; Abcam), MMP-13 (1:1000; ab51072; Abcam), ADAMTS-5 (1:1000; ab231595; Abcam), Bax (1:2000; ab182733; Abcam), Bcl-2 (1:2000; ab182858; Abcam), cleaved caspase-3 (1:500; ab2302; Abcam), LC3 (1:2000; ab192890; Abcam), beclin-1 (1:1000; ab210498; Abcam), p62 (1:1000; ab207305; Abcam), JAK2 (1:1000; ab170718; Abcam), phospho-JAK2 (pJAK2; 1:2000; ab32101; Abcam), STAT3 (1:2000; ab68153; Abcam), phosphor-STAT3 (pSTAT3; 1:2000; ab76315; Abcam), AMPK (1:1000; #12063; Cell Signalling Technology Inc., Danvers, MA, USA), p-AMPK (1:1000; #4186; Cell Signalling Technology Inc.), ERK1/2 (1:1000; ab17942, Abcam), p-ERK (1:1000; ab192591, Abcam), p38 (1:1000; ab31828, Abcam), p-p38 (1:1000; ab47363, Abcam), JNK (1:1000; ab208035, Abcam), p-JNK (1:1000; ab124956, Abcam), Akt (1:1000; #4691; Cell Signalling Technology Inc.), p-Akt (1:2000; #4060; Cell Signalling Technology Inc.), and GAPDH (1:2500; ab9485; Abcam) at 4 °C overnight. After washing with PBS, the membrane was incubated with an HRP-conjugated secondary antibody goat anti-rabbit IgG (1:2000; ab6721; Abcam) at room temperature for 2 h. Development was performed using enhanced chemiluminescence reagents (Beyotime). Protein was detected with Image Acquisition using ImageQuant LAS 4000 (GE Healthcare Life Sciences, Marlborough, MA, USA).

### Immunofluorescence

NPCs were seeded into 24-well plates with sterile coverslips embedded, and cells were cultured to 80% confluence. The medium was removed and washed twice with PBS, and then the cells were fixed in 3.5% formaldehyde at room temperature for 30 min. After washing the cells 3 times with PBS, they were treated with 0.1% Triton X-100 in PBS for 20 min. Subsequently, the cells were incubated with 3% bovine serum albumin and 0.05% Tween for 30 min at 37 °C. After washing with PBS, the cells were incubated with the primary antibody at 4 °C overnight. After washing, cells were treated with a fluorescent secondary antibody (1:500; Abcam) for 20 min at room temperature. Then DAPI (0.1 μg/mL) was added dropwise for 5-10 min. Slides were mounted on glass slides after the anti-fluorescent quencher was added dropwise. Fluorescence images were acquired under a confocal microscope (Leica, Mannheim, Germany).

### Flow cytometry

Annexin V/propidium iodide (PI) apoptosis detection kit (Beyotime) was used to evaluate apoptosis. Briefly, NPCs (1 × 10^6^) were seeded into 6-well plates, and then incubated with Annexin V-fluorescein isothiocyanate and PI for 30 min in the dark. Apoptosis was analysed using flow cytometry (ThermoFisher Scientific).

### The establishment of rat IVDD model

An IVDD model was prepared in 8-week-old SD rats using a fibrous ring puncture method (Jeong et al. 2010). A total of 24 rats were assigned to three groups (*n* = 8 per group): sham group, IVDD group and IVDD + cyanidin group. The rat tail disc (Co4-5) was located on the coccygeal vertebrae. A 26 G puncture needle was used to pierce the entire annulus of the fibre through the skin of the tail. The needle was held in the disc for 1 min. Immediate after the operation, cyanidin was intraperitoneally injected at a dose of 50 mg/kg per day (Jiang et al. 2019). Rats in the control group and the IVDD group were injected with the same dose of physiological saline. Eight weeks after treatment, rats were sacrificed by overdosing 0.1% sodium pentobarbital, tails were harvested and tail disc samples were collected.

### Histopathologic analysis

Tail disc samples were fixed in 4% paraformaldehyde solution for 24 h. The samples were dehydrated with gradient alcohol, cleared in xylene, and embedded in paraffin, and then sectioned (5 μm). Paraffin sections were hydrated and stained in haematoxylin for 5 min. The sections were treated with a 1% hydrochloric acid alcohol solution for 20 s, and then returned to blue in a 1% aqueous ammonia solution. After counter-staining with 1% eosin solution for 5 min, the sections were dehydrated with alcohol by gradient. The sections were transparent in xylene, and then mounted with a neutral gum. A BH-2 microscope (Olympus, Tokyo, Japan) was used for observation.

### Immunohistochemical

After paraffin sections were dewaxed and hydrated, they were incubated in freshly prepared 3% H_2_O_2_ for 10 min at room temperature. Subsequently, the sections were subjected to antigen retrieval by microwave for 20 min. Non-immune animal serum was added dropwise to the sections and blocked at 37 °C for 15 min. Next, the primary antibody was added dropwise to the sections, and incubated in a refrigerator at 4 °C overnight. After washing with PBS, a biotinylated secondary antibody was added dropwise to the sections and incubated at 37 °C for 40 min. Fresh prepared DAB colour development solution was added to the sections for colour development, and terminated in time under a microscope. After the sample was counterstained with haematoxylin staining solution, it was rinsed with distilled water. After dehydration by gradient ethanol, the samples were immersed in xylene for 5 min, and then the tablets were sealed with neutral gum. Five areas were selected to take pictures under the light microscope.

### Terminal deoxynucleotidyl transferase dUTP nick-end labelling (TUNEL) assay

According to the manufacturer's protocol, the ApopTag InSitu apoptosis detection kit (Boster, Wuhan, China) was used to perform an *in situ* TUNEL reaction on the tail disc samples. In brief, paraffin sections were blocked with non-immune animal serum for 15 min after dewaxing, hydration, antigen retrieval, and endogenous peroxidase blocking. TUNEL reaction mixture (5 μL TdT + 50 μL fluorescein-labeled dUTP) was added to the sections and treated in a dark wet box at 37 °C for 1 h. Converter-POD (50 μL) was added dropwise to the sections and processed in a 37 °C dark wet box for 30 min. DAB substrate (Beyotime) (50 μL) was added dropwise to a glass slide and developed at 20 °C for 10-30 min. The sections were stained with DAPI (5 μg/mL) and stained at room temperature for 5 min, and then dehydrated with gradient alcohol, xylene transparent, and neutral gum seals. Apoptotic cells were imaged under a light microscope (magnification, × 400).

### Statistical analysis

All statistical analyses were performed using SPSS 20.0 software (IBM Corp., Armonk, NY, USA), and graphs were generated using GraphPad Prism 5 Software (Graph Pad Software, Inc., La Jolla, CA, USA). Student’s *t*-test was used to analyse protein expression. ANOVA was also performed comparing more than two groups. *p* (two-tailed) < 0.05 were considered statistically significant.

## Results

### Effects of cyanidin on cell viability

**Figure 1.**
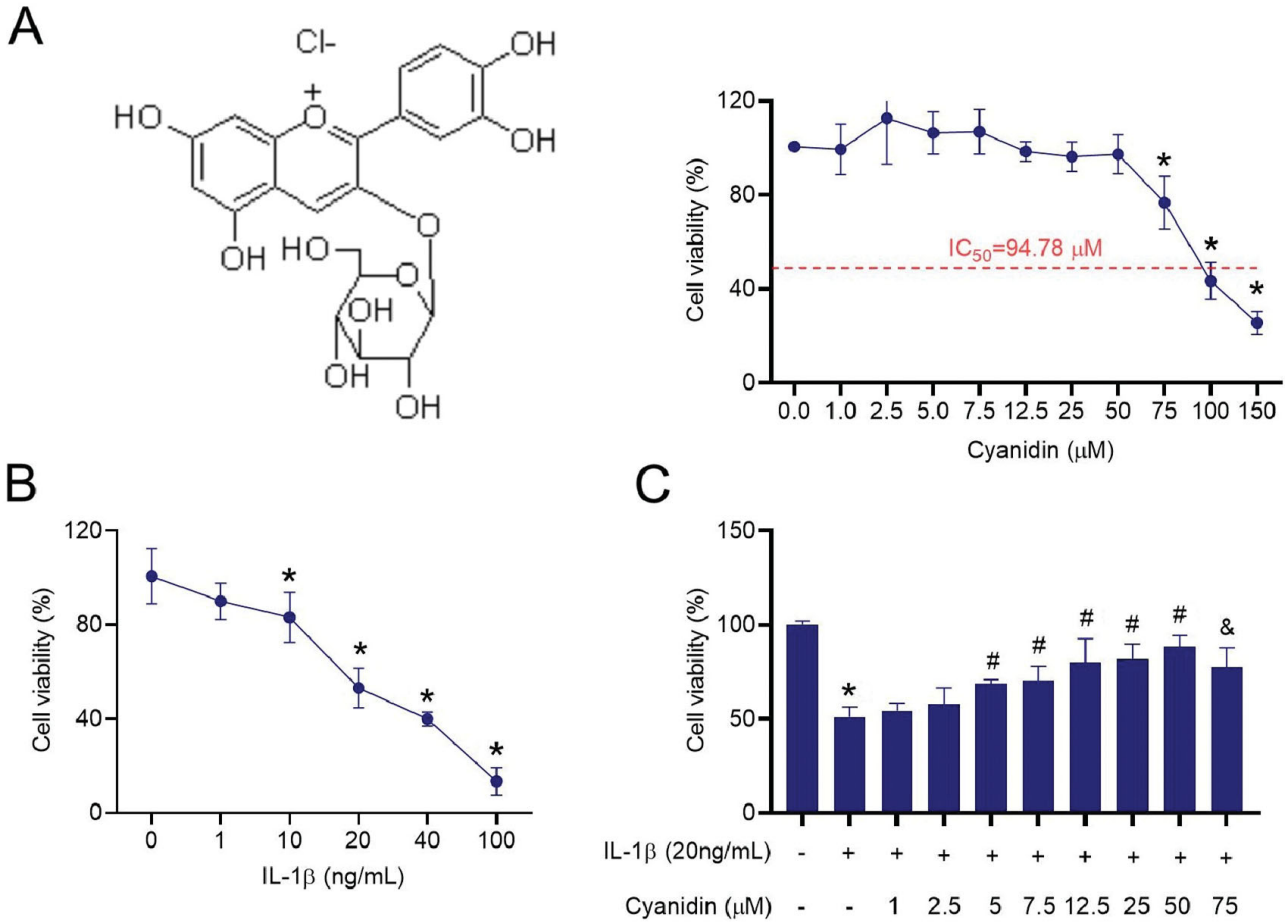
Effects of cyanidin and IL-1β on cell viability. The cytotoxic effect of cyanidin (A) and IL-1β (B) on human NPCs was measured at various concentrations for 24 h using a MTT assay. (C) The effect of cyanidin pre-treatment on the viability of IL-1β-induced NPCs was measured by a MTT assay. *p < 0.05 vs. control; ^#^*p* < 0.05 vs. NPCs treated with IL-1β only; ^&^*p* < 0.05 vs. NPCs treated with IL-1β and cyanidin.

NPCs were treated with different concentrations of cyanidin for 24 h. The MTT assay was subsequently used to analyse the toxic effect of cyanidin on NPCs. As shown in Figure 1(A), cyanidin did not affect the cell viability of NPCs at concentrations below 50 μM, while cyanidin significantly reduced the viability of NPCs at concentrations higher that 75 μM. The IC_50_ of cyanidin for NPCs was 94.78 µM. Moreover, IL-1β can be used to establish IVDD *in vitro* (Deng et al. 2017). We found that when the concentration of IL-1β was greater than 10 ng/mL, the activity of NPCs was decreased, and the inhibition was enhanced with increasing concentration (Figure 1(B)). Hence, we used 20 ng/mL of IL-1β for subsequent experiments (Deng et al. 2017). In addition, pre-treatment with cyanidin prevented cell death induced by IL-1β, especially at 50 μM (Figure 1(C)). Hence, 25 and 50 μM cyanidin were selected in subsequent experiments.

### Cyanidin mitigated ECM synthesis and degradation and apoptosis in IL-1β-induced NPCs

**Figure 2.**
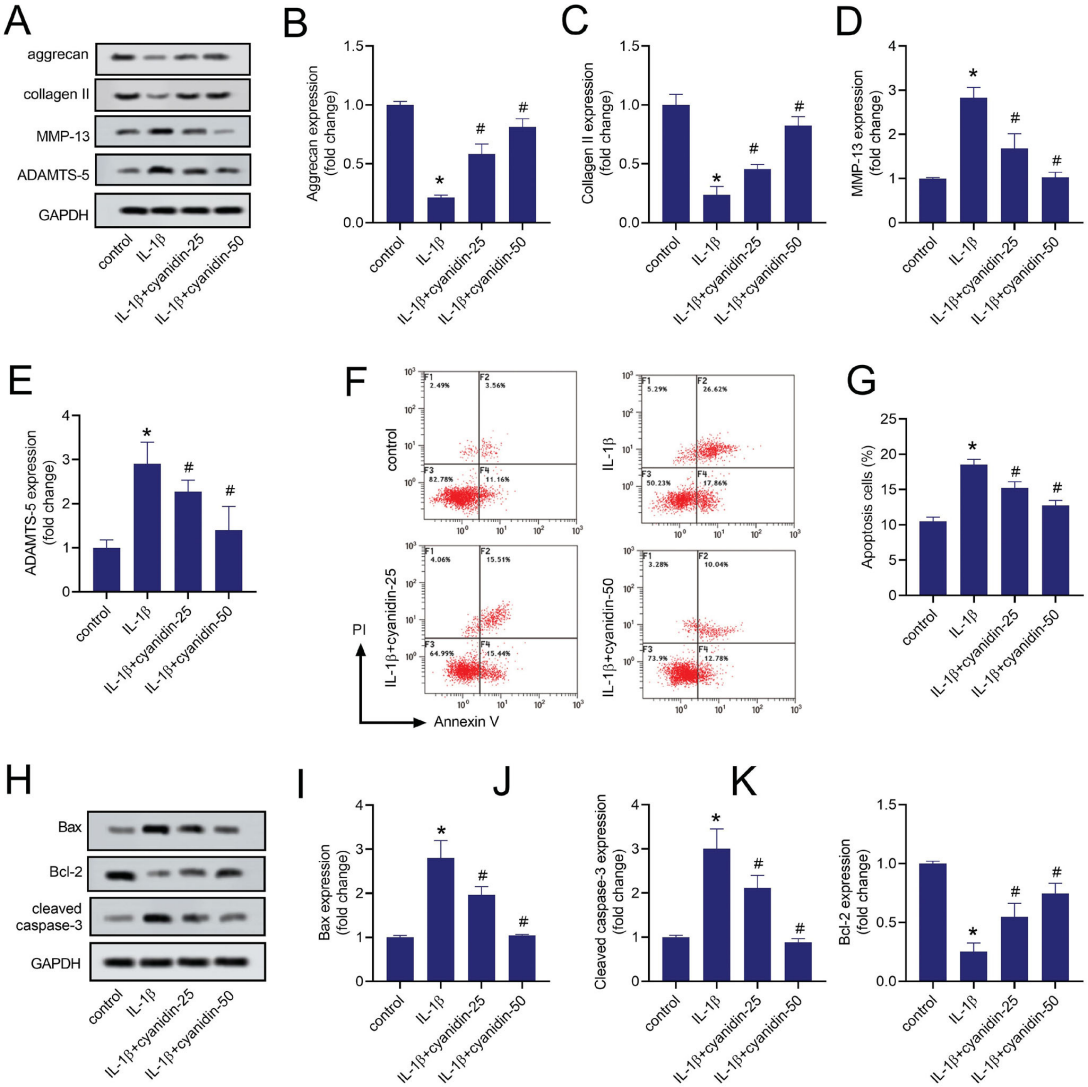
Cyanidin mitigated ECM synthesis and degradation and apoptosis in IL-1β-induced NPCs. (A–E) The expression of aggrecan, collagen II, MMP-13 and ADAMTS-5 in NPCs were analysed by western blot assays. (F,G) Flow cytometry was used to detect the apoptosis of NPCs. (H–K) The expression of Bax, Bcl-2 and cleaved caspase-3 in NPCs were analysed by western blot assays. *p < 0.05 vs. control; ^#^*p* < 0.05 vs. NPCs treated with IL-1β only.

Western blot assay results also showed downregulated expression of aggrecan (Figure 2(A,B)) and collagen II (Figure 2(A,C)), and elevated expression of MMP-13 (Figure 2(A,D)) and ADAMTS-5 (Figure 2(A,E)) in IL-1β- induced NPCs. However, cyanidin treatment promoted the expression of aggrecan and collagen II in cells induced by IL-1β, while inhibiting the expression of MMP-13 and ADAMTS-5 (Figure 2(A–E)). We further analysed the effect of cyanidin treatment on the apoptosis of IL-1β-induced NPCs. Flow cytometry showed that IL-1β treatment induced apoptosis in NPCs, while cyanidin treatment reduced the number of apoptotic cells in IL-1β-induced NPCs (Figure 2(F,G)). Simultaneously, western blots showed that cyanidin treatment restrained the expression of Bax and cleaved caspase-3, while promoting the expression of Bcl-2 protein (Figure 2(H–K)). These results suggested that cyanidin attenuated ECM degradation and apoptosis of degenerative NPCs.

### Cyanidin accelerated autophagy in IL-1β-induced NPCs

**Figure 3.**
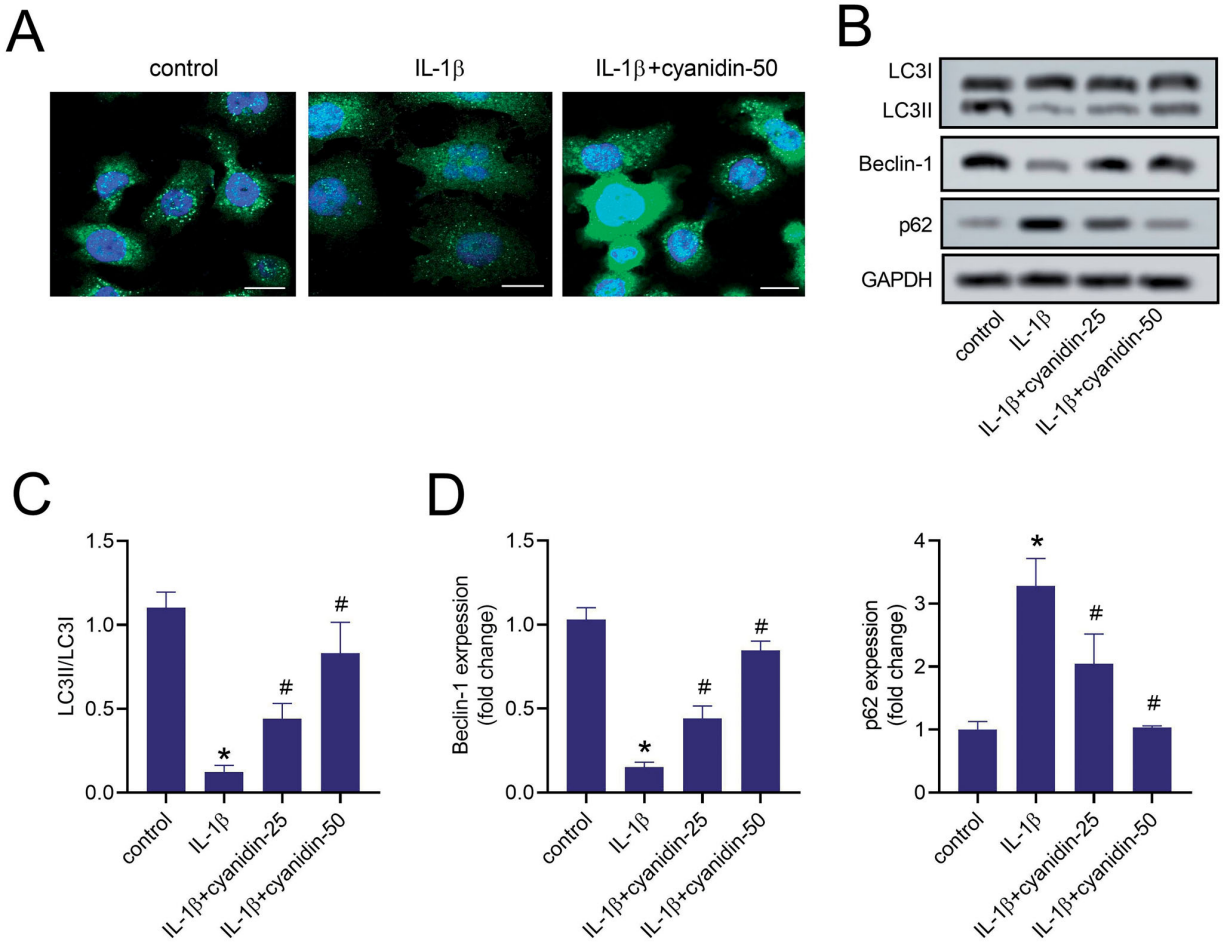
Cyanidin accelerated autophagy in IL-1β-induced NPCs. (A) LC3-positive autophagosomes was observed by immunofluorescence staining (Bar = 25 μm). (B–E) The expression of autophagy related proteins LC3, beclin-1 and p62 were determined by western blots. *p < 0.05 vs. control; ^#^*p* < 0.05 vs. NPCs treated with IL-1β only.

To confirm whether cyanidin induced autophagy in NPCs, we determined the induction of autophagy by localising the autophagosome-specific protein LC3. As shown in Figure 3(A), LC3 puncta in cytoplasm of NPCs were decreased with IL-1β administration, while pre-treatment with cyanidin obviously reversed this effect. Simultaneously, western blot assay results showed that the ratio of LC3II/LC3I and the expression of beclin-1 in IL-1β-induced NPCs were significantly reduced, and could be reversed by cyanidin pre-treatment (Figure 3(B–E)). We found that cyanidin pre-treatment markedly reduced the expression of p62 in IL-1β-induced NPCs (Figure 3(B–E)). These data indicated that cyanidin promoted autophagy in degenerative NPCs *in vitro*.

### Autophagy enhanced inhibitory effects of cyanidin on the apoptosis and ECM degradation of IL-1β-induced NPCs

**Figure 4.**
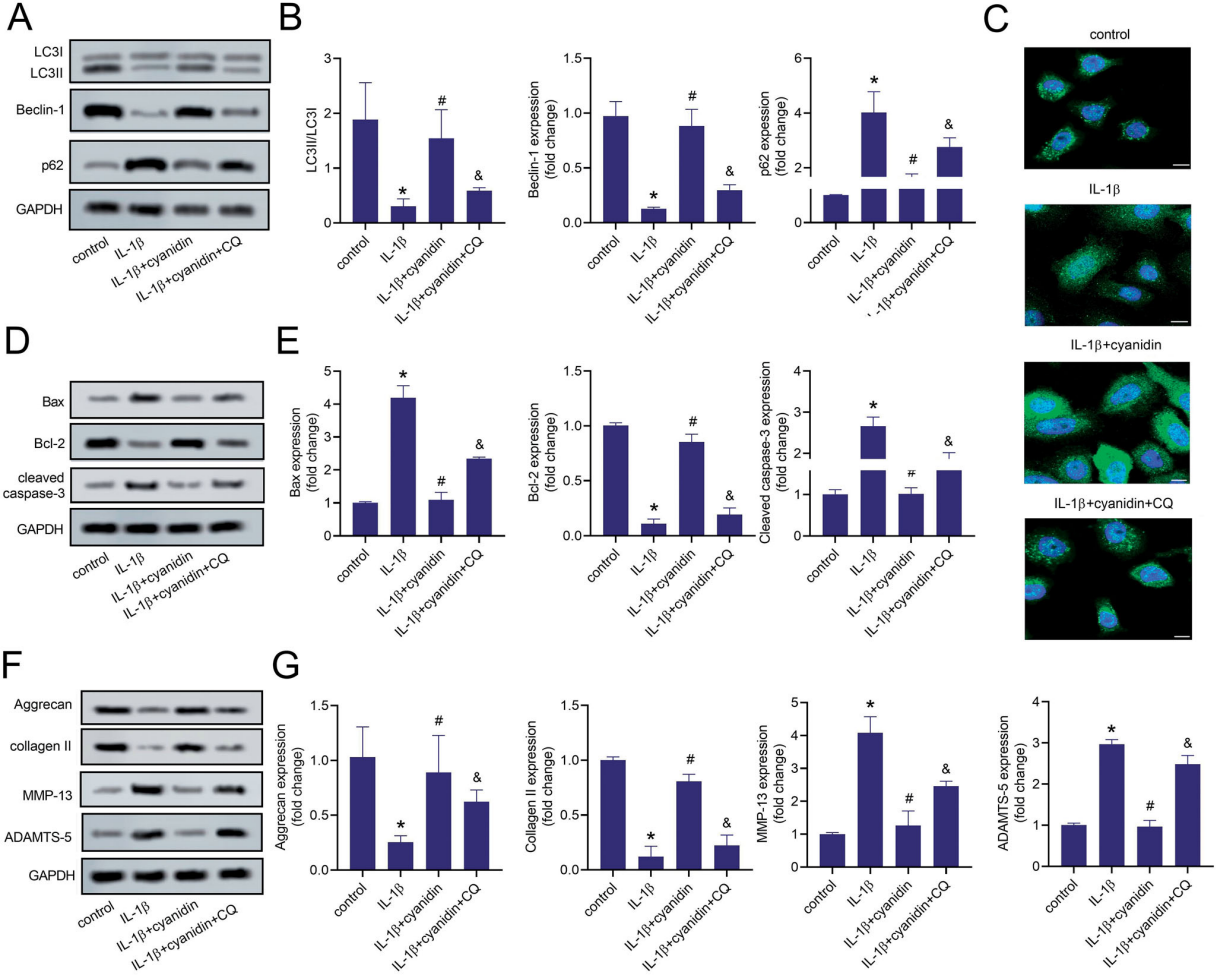
Autophagy enhanced the inhibitory effect of cyanidin on IL-1β-induced NPCs apoptosis and ECM degradation. (A,B) The expression of autophagy related proteins LC3, beclin-1 and p62 were determined by western blots. (C) LC3-positive autophagosomes was observed by immunofluorescence staining (Bar = 25 μm). (D,E) Western blots were used to analyse the expression of Bax, Bcl-2, cleaved caspase-3. (F,G) The expression of collagen II, aggrecan, MMP-13 and ADAMTS-5 were analysed using western blot assays. *p < 0.05 vs. control; ^#^*p* < 0.05 vs. NPCs treated with IL-1β only; ^&^*p* < 0.05 vs. NPCs treated with IL-1β + cyanidin.

To explore the role of autophagy in the apoptosis and ECM degradation of NPCs, IL-1β-induced NPCs were pre-treated with CQ, the autophagy inhibitor, before cyanidin treatment. Compared with the IL-1β + cyanidin group, CQ pre-treatment markedly decreased the LC3II/LC3I ratio, as well as the expression of beclin-1, while increasing the expression of p62 (Figure 4(A,B). Immunofluorescence staining also showed that LC3 puncta in the cytoplasm of IL-1β-induced NPCs were increased with cyanidin treatment, which was abolished by CQ pre-treatment (Figure 4(C)). Additionally, CQ pre-treatment obviously attenuated the effects of cyanidin on the expression of apoptotic proteins, Bax, Bcl-2 and cleaved caspase-3, in IL-1β-induced NPCs (Figure 4(D,E)). Moreover, CQ reversed the effects of cyanidin on the expression of collagen II, aggrecan, MMP-13 and ADAMTS-5 (Figure 4(F,G)). These results suggested that autophagy was involved in the effect of cyanidin on the apoptosis and ECM degradation of NPCs.

### Inhibition of the JAK2/STAT3 pathway was important for the protective effects of cyanidin on IL-1β-induced NPCs

**Figure 5.**
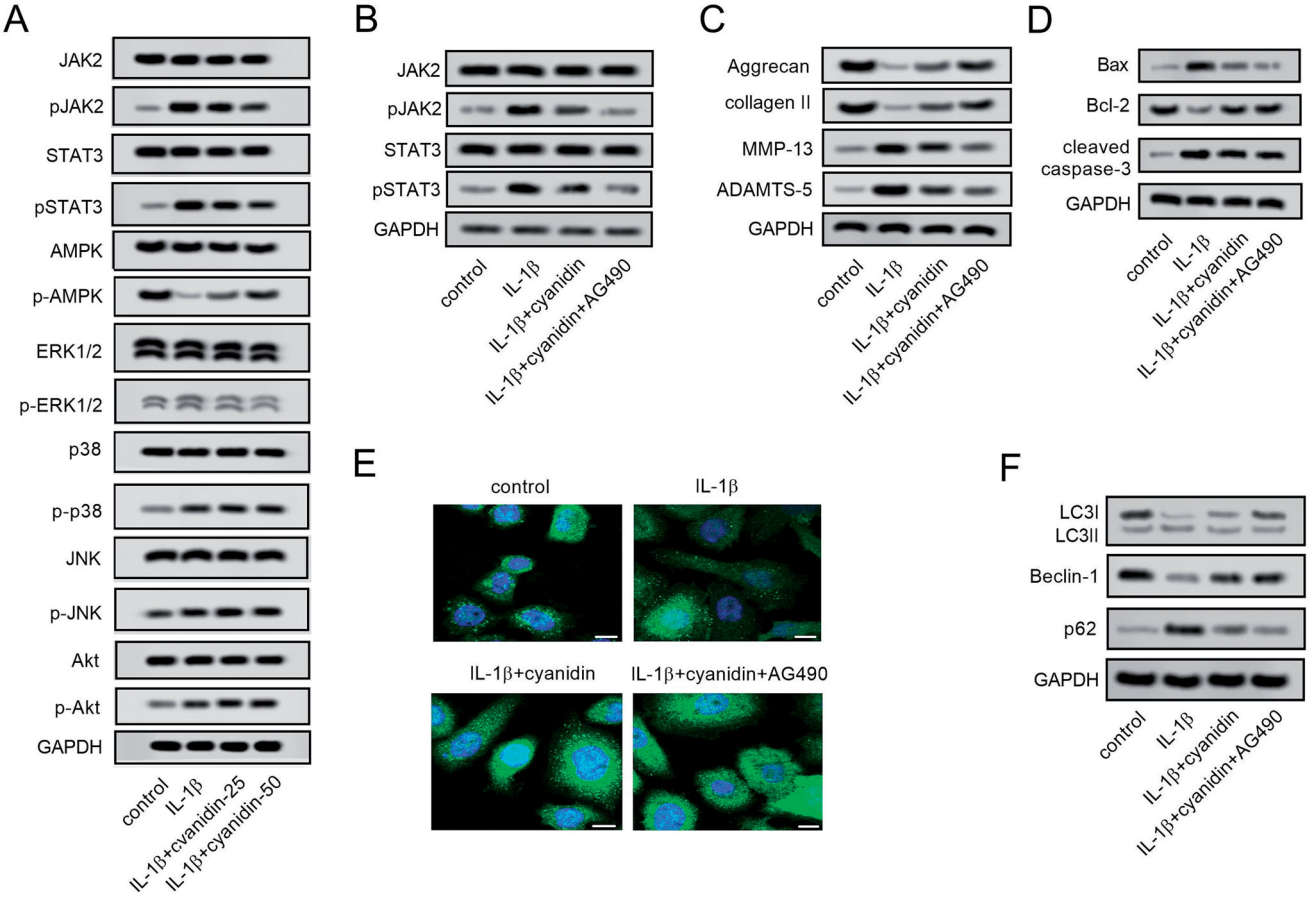
Inhibition of JAK2/STAT3 pathway was important for the protective effects of cyanidin on IL-1β-induced NPCs. (A) IL-1β-induced NPCs were treated with different concentration of cyanidin, and the related proteins of the JAK2/STAT3 pathway, the AMPK pathway, the MAPK pathway and the PI3K/Akt pathway were analysed using western blot assays. IL-1β-induced NPCs were treated with cyanidin and AG490, and the expression of JAK2, p-JAK2, STAT3, and p-STAT3 (B), ECM-related proteins collagen II, aggrecan, MMP-13 and ADAMTS-5 (C), apoptosis-related proteins Bax, Bcl-2 and cleaved caspase-3 (D) were analysed by western blots. (E) Immunofluorescence staining was used to observe LC3 positively stained autophagosomes (Bar = 25 μm). (F) The expression of autophagy-related proteins LC3, beclin-1 and p62 were detected by western blots. *p<0.05, **p<0.01.

Cyanidin treatment down-regulated the expression of p-JAK2 and p-STAT3 in IL-1β-induced NPCs (Figure 5(A)). Besides, IL-1β reduced the phosphorylation of AMPK, which was reversed by cyanidin treatment. However, cyanidin barely affected the activities of MAPK/ERK1/2 pathway and PI3K/Akt pathway. Moreover, AG490 enhanced the inhibition of JAK2 and STAT3 protein phosphorylation by cyanidin treatment (Figure 5(B)). Additionally, the effects of cyanidin on synthesis and degradation of ECM, apoptosis, as well as autophagy of IL-1β-induced NPCs were also enhanced by AG490 (Figure 5(C–F)). These results revealed that the protective effect of cyanidin on degenerated nucleus pulposus cells may be related to its inhibitory effect on the JAK2/STAT3 signalling pathway.

### Cyanidin mitigated disc degeneration in rats *in vivo*

**Figure 6.**
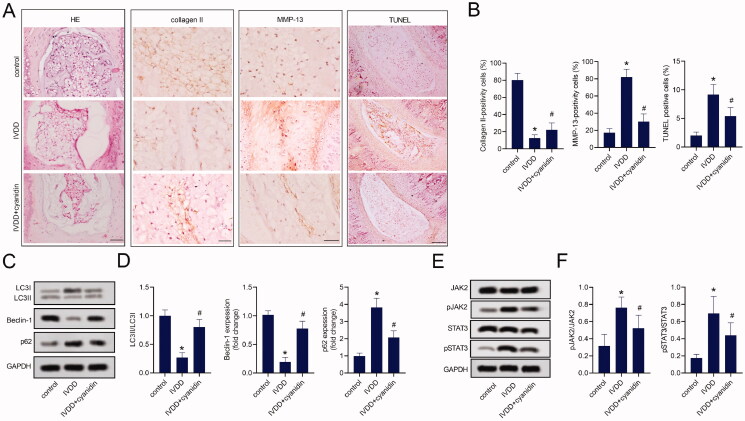
Cyanidin mitigated disc degeneration in rats *in vivo*. (A) The pathological damage of the nucleus pulposus was observed by HE staining (Bar = 100 μm), the expression of collagen II in the disc samples was analysed by immunohistochemistry at 8 weeks post-surgery (Bar = 50 μm), and TUNEL assay was used to analyse the apoptosis of the nucleus pulposus in the tail of IVDD model rats (Bar = 200 μm). (B) The quantification of immunohistochemistry and TUNEL assays. (C,D) Western blot assays were used to analyse the expression of LC3, beclin-1 and p62 in the nucleus pulposus of the tail vertebra of IVDD model rats. (E,F) Expression of JAK2/STAT3 pathway related proteins in disc samples were detected by western blot assays. *N* = 8. *p < 0.05 vs. control; ^#^*p* < 0.05 vs. IVDD.

In order to further explore the effect of cyanidin on the improvement of IVDD, a surgical IVDD model was established, followed by intraperitoneal injection of 50 mg/kg of cyanidin or saline for 8 weeks. Then, tail disc nucleus pulposus tissues of IVDD rats were analysed using HE staining (Figure 6(A)). The results showed that the number of NPCs in the intervertebral disc tissues of the IVDD group was decreased, and the arrangement of annulus fibrous was disordered and ruptured. However, compared to the IVDD group, cyanidin intervention effectively alleviated the degeneration of NPCs as well as the disorganisation of annulus fibrous. Immunohistochemical analysis displayed (Figure 6(A,B)) that reduced collagen II expression and increased MMP-13 expression in nucleus pulposus tissues of IVDD rats were reversed by cyanidin treatment. TUNEL also illustrated that cyanidin treatment improved the occurrence of apoptosis in the nucleus pulposus tissue of IVDD rats (Figure 6(A,B)). Additionally, western blot assays demonstrated that compared with the control group, the LC3II/LC3I ratio and beclin-1 expression were decreased, while p62 expression was increased in the nucleus pulposus tissues of IVDD rats. However, cyanidin treatment promoted autophagy in the nucleus pulposus tissue of IVDD rats (Figure 6(C,D)). Moreover, cyanidin treatment significantly inhibited the phosphorylation of JAK2 and STAT3 proteins in degenerated nucleus pulposus of IVDD rats (Figure 6(E,F)). Collectively, these data suggested that cyanidin alleviated IVDD.

## Discussion

Cyanidin has been shown to have therapeutic potential for a variety of diseases including osteoarthritis, ischemia-reperfusion injury, and respiratory diseases (Xu et al. 2015; Li et al. 2018; Jiang et al. 2019). However, the role of cyanidin in IVDD is unknown. The present study found that cyanidin inhibited the apoptosis and ECM degradation of NPCs induced by IL-1β, which might be related to its induction of autophagy. Consistently, cyanidin attenuated the degeneration of intervertebral discs in IVDD rats. More importantly, the protective effect of cyanidin on IVDD might be achieved by inhibiting the JAK2/STAT3 pathway.

Aggrecan and collagen II are the main components of the nucleus pulposus ECM that play important roles in maintaining the elasticity and osmotic pressure of the disc (Xu et al. 2019). IVDD is often accompanied by degradation of the ECM, which is caused by the imbalance in the synthesis and catabolism of the ECM (Tao et al. 2016). Catabolism is mainly controlled by proteolytic enzymes, of which MMPs and ADAMTSs play the key role (Wang et al. 2015). Besides, the decrease in the number of NPCs caused by excessive apoptosis plays a pivotal role in the pathogenesis of IVDD (Zhao et al. 2017). Factors such as mechanical load, nutritional deficiencies, hyperglycaemia, and pro-inflammatory factors (TNF-α, IL-1β) can induce apoptosis in NPCs. In contrast, anti-IVDD factors, such as resveratrol, platelet-derived growth factor (PDGF), transforming growth factor-β1 (TGF-β1), reduce IVDD by inhibiting NPCs apoptosis (Li et al. 2008). Inhibiting the apoptosis of NPCs and promoting the synthesis of ECM components to inhibit the initiating factors of IVDD are expected to become the key to treat IVDD (Cai et al. 2016; Chen et al. 2018).

Cyanidin is an anthocyanidin compound that has various biological functions such as anti-inflammatory and antioxidant (Daveri et al. 2018). Noteworthy, research shows that cyanidin exhibited a chondrogenesis stimulating effect by upregulating the expression of aggrecan and col2a1 (Saulite et al. 2019). Further, cyanidin can reduce the degradation of aggrecan and collagen II in human osteoarthritis chondrocytes by inhibiting IL-1β-induced inflammatory response (Jiang et al. 2019). Moreover, the regulatory effect of cyanidin on cell apoptosis has also been extensively studied. Qian et al. (2018) demonstrated that cyanidin can down-regulate Bcl-2 homologous antagonist/killer and up-regulate Bcl-2 to reduce cisplatin-induced apoptosis. Furthermore, cyanidin attenuates apoptosis mediated by mitochondrial apoptotic pathway and ER stress pathway by reducing oxidative stress (Thummayot et al. 2018). Consistent with previous research, we also found that cyanidin promoted the expression of aggrecan and collagen II in cells induced by IL-1β, while inhibited the expression of MMP-13 and ADAMTS-5. In addition, cyanidin treatment reduced the number of apoptotic cells in IL-1β-induced NPCs. Additionally, cyanidin treatment restrained the expression of Bax and cleaved caspase-3, while promoting the expression of Bcl-2 protein. Taken together, these results revealed that cyanidin could improve IVDD by inhibiting ECM degradation and NPCs apoptosis, which may have therapeutic potential in IVDD treatment.

Autophagy has become a topic of intense research in recent years. Increasing evidence shows that autophagy is widely involved in the development of many degenerative diseases, including osteoarthritis (Zhong et al. 2019), neurodegenerative diseases (Wang et al. 2018) and IVDD (Li et al. 2020). Autophagy is also considered to be an important pathway for regulating ECM metabolism in the disc. Wang et al. (2016) found that resveratrol inhibits ECM degradation by increasing TNF-α-induced MMP-3 expression by increasing autophagy levels of NPCs. Zhang et al. (2018) reported that downregulation of IL-1-induced MMP-13 expression by activating autophagy in rat NPCs inhibits ECM degradation. Therefore, autophagy may help prevent ECM degradation under inflammatory conditions. In addition, as an independent type II programmed cell death process, there is a close relationship between autophagy and apoptosis (Dang et al. 2015; Fernández et al. 2015). For example, the autophagy-related protein Atg5 can cause autophagy death by interacting with Fas-related death domain proteins (Yousefi et al. 2006), while Bcl-2, the apoptosis-regulating protein, can bind to beclin-1, the autophagy regulator, to regulate autophagy (Luo and Rubinsztein 2010). Jiang et al. (2014) found that silent information regulation 2 homolog1 (SIRT1) can inhibit the apoptosis of NPCs by up-regulating autophagy Furthermore, SIRT1 agonist resveratrol can improve the apoptosis of mouse degenerative discs and promote the synthesis of collagen II (Xia et al. 2015). These studies suggested that autophagy may be a protective mechanism for disc degeneration. This study also found that the number of autophagosomes in IL-1β-induced NPCs was significantly reduced, while the ratio of LC3II/LC3I and the expression of beclin-1 were decreased, suggesting a low-level of autophagy in degenerating NPCs. Besides, the autophagy inhibitor CQ reversed the effects of cyanidin on the apoptosis and ECM degradation of IL-1β-induced NPCs, manifesting that autophagy might be involved in the effect of cyanidin on the apoptosis and ECM degeneration of NPCs. Collectively, we speculated that the inhibitory effect of cyanidin on NPCs apoptosis and ECM degradation might be achieved by inducing autophagy.

The molecular mechanisms involved in autophagy are extremely complex, in which the AMPK signalling pathway (Yang et al. 2019), the PI3K/Akt pathway (Deng et al. 2017) and the MAPK/ERK1/2 pathway (Wang et al. 2019) are relevant. Recent studies have also found that the JAK2/STAT3 signalling pathway is involved in the regulation of autophagy (Feng et al. 2014). Among them, AMPK activation is a key factor related to autophagy in cells (Herzig and Shaw, 2018). The present study showed that IL-1β reduced the phosphorylation of AMPK, which was reversed by cyanidin treatment, suggesting that cyanidin induce autophagy in IL-1β-treated NPCs. However, cyanidin barely affected activities of the MAPK/ERK1/2 pathway and the PI3K/Akt pathway. The abnormal activation of the JAK2/STAT3 pathway has been shown to be involved in a variety of pathophysiological processes including apoptosis and autophagy (Du et al. 2019). Research shows that inhibition of STAT3 can lead to transformation of LC3, formation of autophagosomes, and degradation of p62, which are all hallmarks of the autophagy program (Shen et al. 2020). In contrast, STAT3 overexpression induces downregulation of this pathway (Shen et al. 2012). Furthermore, inhibition of the JAK2/STAT3 pathway in human NPCs can delay IVDD by inhibiting NPCs apoptosis and ECM degradation (Suzuki et al. 2017; Zhou et al. 2017). Moreover, activation of the JAK2/STAT3 pathway plays an important role in the progression of IVDD (Miao and Zhang 2015). The present study also illustrated that cyanidin treatment down-regulated the expression of p-JAK2 and p-STAT3 in IL-1β-induced NPCs, suggesting that inhibition of the JAK2/STAT3 pathway might be related to the protective effects of cyanidin on the degenerative NPCs. Moreover, AG490, the antagonist of JAK2, enhanced the protective effects of cyanidin on degenerated NPCs. Therefore, the anti-ECM degradation, anti-apoptosis, and autophagic effects of cyanidin on degenerated nucleus pulposus cells may be related to its inhibitory effect on the JAK2/STAT3 signalling pathway.

However, our research also has some limitations. Considering the anti-inflammatory and antioxidative stress effects of cyanidin, it is necessary to study the protective effects of cyanidin on mitochondrial dysfunction in the future. Furthermore, additional pharmacological studies are required to elucidate the additional mechanisms by which cyanidin improves IVDD.

## Conclusions

Cyanidin inhibited the ECM degradation and apoptosis of IL-1β-induced NPCs. Additionally, autophagy was involved in the effects of cyanidin on the apoptosis and ECM degradation of NPCs. According to the *in vivo* study, we inferred that cyanidin improved IVDD by inhibiting activation of the JAK/STAT3 pathway. In conclusion, cyanidin improved the function of NPCs in IVDD *in vitro* and *in vivo* by promoting autophagy, inhibiting cell apoptosis and ECM degradation via negatively regulating the JAK/STAT3 signalling pathway. These findings further reveal the specific mechanism by which cyanidin exerts its protective effects on IVDD, indicating that cyanidin can prevent or treat IVDD.

## Data Availability

All data generated or analysed during this study are included in this published article.
